# The Development and Validation of the LIMPRINT Methodology

**DOI:** 10.1089/lrb.2018.0081

**Published:** 2019-04-22

**Authors:** Christine Moffatt, Peter Franks, Vaughan Keeley, Susie Murray, Gregoire Mercier, Isabelle Quéré

**Affiliations:** ^1^ School of Social Sciences, Nottingham Trent University, Nottingham, United Kingdom.; ^2^ Montpellier Medecine Vasculaire, EA2992, Universite Montpellier I, CHU Saint Eloi, Montpellier, France.; ^3^ Copenhagen Wound Healing and Lymphoedema Centre, Bisperberg University Hospital, Copenhagen, Denmark.; ^4^ Center for Research & Implementation of Clinical Practice, London, United Kingdom.; ^5^ Royal Derby Hospital, Derby, United Kingdom.

**Keywords:** LIMPRINT, epidemiology, chronic edema, primary lymphedema, lymphoedema, secondary lymphedema

## Abstract

The acronym Limprint stands for Lymphedema IMpact and PRevalence INTernational and was run under the auspices of the International Lymphedema Framework (ILF), a charity dedicated to improving provision of care globally. The primary aim was to identify the number of people with chronic edema (chronic edema present for >3 months and due to a range of underlying etiologies and associated risk factors) within diverse health services in nine participating countries and to determine its impact using validated methods. An international protocol and sampling framework, online data capture system, and standard operating procedures were adopted. An international consensus was used to create a core data tool that covered 13 domains. Specialist data on demographics and disability, details of swelling, wounds, cancer, and health-related quality of life were also available for sites. The study protocol was designed to allow flexibility in the types of studies undertaken within complex health care systems. All cases were confirmed using the modified pitting test. Sensitivity and specificity for this method were determined in Japanese and European populations. Following confirmation of a chronic edema case, Lymphologists defined whether it was a primary of a secondary form. The study was designed to provide robust evidence that chronic edema is an important and unrecognized public health problem in health services with significant morbidity. Without evidence of the size and complexity, it will remain considered a rare phenomenon and people affected will be denied access to appropriate treatment that would allow them to have fulfilled and productive lives.

## Background

### International Lymphedema Framework and National Lymphedema Frameworks

Chronic edema remains a worldwide issue. The LIMPRINT© study is at the core of the guiding principles of the International Lymphedema Framework (ILF).^1^ The ILF is a U.K.-based charity whose aim is to improve the management of chronic edema and related disorders worldwide through research and the sharing of expertise and resources. The ILF comprises member countries that subscribe to the ideals of the ILF and have each developed their own independent National Lymphedema Framework (NLF)—a partnership of stakeholders (including: clinicians, academics, patients, lymphology organizations and other relevant groups) dedicated to improving chronic edema care. An important part of the ILF strategy is to support countries in acquiring and presenting data that will:

- Establish the size of the problem of chronic edema
- Provide the basis for evidence-based practice
- Be used to facilitate improved reimbursement

### Rationale

This article describes the methods adopted in the development and validation of the LIMPRINT study.

### LIMPRINT

The acronym LIMPRINT stands for Lymphedema IMpact and PRevalence- INTernational Lymphedema Framework and presents the aim of the study, which is “To determine the impact and prevalence of chronic edema within health services at a national and international level using a common methodology.”

### LIMPRINT project overview

LIMPRINT© was a two-phased project. Phase 1 was conducted between June 2013 and June 2014 with the development and validation of the methods within two large pilot projects. Phase II involved roll out across all sites (9 countries and 40 sites) from June 2014 until August 2017. Table 1 indicates the stages of the project plan.

**Table 1.** Project Plan

*Phase 1: Development and validation*
*Stages*
1. Agreement of the research methodologies that could be developed for international use in complex health care systems
2. Literature review of prevalence study methods and epidemiology
3. Development of questionnaires for the core and module specialist tools
4. Development and validation of a classification for chronic edema
5. Interrater reliability studies (pitting test and classification of chronic edema in different populations)
6. Construction and testing of the online database
7. Development of an international protocol and sampling frameworks
8. Development of a support manual and educational tools
9. Establishment of quality control mechanisms

*Phase 2: Main study*
*Stages*
1. Epidemiology studies undertaken in all participating sites
2. Data quality monitoring
3. Data analysis and reporting

## Detailed Project Development

The stages of the LIMPRINT study are described below. While presented as sequential stages, many of them ran concurrently over the development phase.

### Phase 1. Stage 1. Agreement of the research methodologies

The approach taken for the LIMPRINT study was decided during an international ILF conference in Montpellier in 2012.^2^ Key stakeholders from frameworks and methodologists discussed the strengths and limitations of different epidemiological approaches within complex and varied health care systems. While it was acknowledged that large population-based studies were the gold standard, this was not a realistic option for the following reasons:

• Lack of international resources
○ Census or reliable general population data from which to draw random samples.
○ Insurance data for random sampling in public health care systems
• Large populations would require screening to generate a modest sample size
• International coding systems do not exist to interrogate existing data sets
• Bias in population and health service data sets

The core working group defined the key priority as “The need to uncover the hidden burden of chronic edema on health services.” A method of case ascertainment previously used in the United Kingdom was adopted as the most flexible approach using the following public health definition of chronic edema.^3,4^:

“Chronic edema is a broad term used to describe edema, which has been present for more than three months and involves one or more of the following areas: limbs, hands/feet, upper body (breast/chest wall, shoulder, back), lower body (buttocks, abdomen), genital (scrotum, penis, vulva), head, neck, or face.”^3^

### Primary aim and secondary objectives

The primary aim of LIMPRINT was “To determine the number of people within health services suffering from chronic edema and its impact upon them.” Many of these people would not have been previously identified or be receiving treatment before screening during the study.

Secondary objectives were defined to fulfill the scope of the project and were used to determine the study methods and the modular data collection tools (Table 2).

**Table 2.** Primary and Secondary Aims of LIMPRINT

*Primary aim*
To determine the prevalence and functional impact of chronic edema within health services at a national and international level using modular epidemiological tools.

*Secondary objectives*
• To identify, using a case ascertainment questionnaire (CORE TOOL), all patients within defined health services within participating countries, who currently suffer from chronic edema of longer than 3 months duration.
• To determine the impact of chronic edema on the lives of patients using questionnaires as follows:
○ Demographic and disability assessment
○ Health-related quality of life
○ Details of swelling
○ Wound assessment
○ Cancer assessment
• To estimate the proportion of patients with chronic edema who also have a wound in the same anatomical area

The project aimed to facilitate the following types of studies within each country:

- Facility-based prevalence studies: for example, a general hospital or nursing home
- Geographically based prevalence: patients identified in all health services within a defined area
- Specific patient populations: for example, specialist lymphedema services, wound care clinics
- In-depth evaluation of a random sample of patients (within facility- and geographically based studies) for example, to classify type of chronic edema

### Study infrastructure support and patient involvement

An international steering group of relevant experts, including lymphologists, epidemiologists, and statisticians met quarterly throughout the project to ensure project delivery. A separate data monitoring committee was available to answer any questions relating to data issues.

Individual patients and patient organizations within the national frameworks were involved in all aspects of the study, which conformed to current guidance for patient and carer participation (INVOLVE guidelines).^5^

All countries and sites used the international study protocol and conducted the research in accordance with their current ethical and research governance regulatory frameworks and the International Declaration of Helsinki.^6^ All sites complied with standard operating procedures on conducting the study and quality control mechanisms.

### Patient inclusion and exclusion criteria

The study patient inclusion and exclusion criteria are defined in Table 3.

**Table 3.** Inclusion and Exclusion Criteria

*Inclusion criteria*
All ages (data on children only available from specialist services).
Has chronic edema of longer than 3 months.
Is able to understand the study as set out in the information sheet.
Is able to give informed consent, that is, *(gives explicit consent for their data to be transferred into an international data base).*

*Exclusion criteria*
Is unwilling or unable to participate for whatever reason.
Is receiving end-of-life care.
When considered to not be in the patient's best interest (decided by the lead clinician managing their care).

## Methods

To comply with the overall study, the presence and chronicity of edema was confirmed by two methods before chronic edema was judged to be present. This screening test was undertaken at each participating site by staff trained in the data collection methods. In specialist lymphedema services, all patients had been assessed by lymphologists before data collection and the classification of the chronic edema defined. Further description of the methods required in different health care settings is defined below.

*Confirmation of chronic edema* was based on the following two factors:

- First, the existence of edema was determined based on an observational “Pitting Edema Test,” which is widely used in clinical practice and been shown to be valid and reliable.^7^ The pitting test was carried out by pressing the thumb into the site of the swelling for 10 seconds. If a pit remained upon removal of pressure, then pitting edema was judged to be present. The presence of edema was tested in all body parts, using a standard protocol, including the upper and lower limbs, trunk, face, and neck.
- Second, edema was judged to be chronic if it had been present for 3 months or more. This factor was determined on the basis of feedback received from the patient, or if this was not possible, from carers and clinicians who have known the person for at least 3 months.
- In community nursing home studies in the United Kingdom, additional questions were included to ensure that the clarification of chronic edema was correct.

### Phase 1. Stage 2. Literature review of prevalence study methods

A literature review of the current evidence had been undertaken under the leadership of the American Lymphedema Framework Project (ALFP).^8^ This confirmed the lack of evidence available and informed the design of the study and the methods used for data collection.

### Phase 1. Stage 3. Development of the core and module data collection methods

The study design included a core tool, which was completed on all patients irrespective of the type of study. Additional tools were developed for use in more complex projects. The outline of the tools, the methods of use, and the data they provide are outlined in Table 4.

**Table 4.** Tools, Methods, and Data Delivered

*Tool*	*Method*	*Deliverables*
Core tool		
Core case ascertainment questionnaire.	Questionnaire completed by health care professionals	Profile of patients with chronic edema
Identification of patients with chronic edema in health care systems	Chronic edema confirmed by pitting test and confirmation of history of chronic edema >3 months	Age/gender/duration and site of chronic edema
		Subjective control of swelling
		Level of obesity
		Mobility
Used in all studies		Relevant comorbidities
		Classification of cause of chronic edema
		Previous treatment
		Cellulitis history
		Presence of a wound
		Access to treatment
Module tools		
Assessment of functional impact.	Data were gathered from patients using clinical assessment and interviews, either by self-completion or completion by a health professional.	The Module Tools collected data that looked in more depth at factors affecting the life of the patient as well as care delivery, such as the impact on discharge from hospital or accessing appropriate care, including:
		Personal details, including living status, educational attainment, employment status
Demographics and Disability assessment (WHODAS 2.0)		Details of swelling
Quality-of-life assessment (LYMQOL+EQ-5D+ LFSQQ)		Mobility status
Details of swelling		Quality of life
Wound assessment		Impact of cancer
Cancer details		Type and impact of wound
		Factors affecting delivery of care and discharge
		Resource use

### Development of the core tool

The development of the core tool followed a consultative approach with an International expert panel and eight participating NLFs. The resulting core tool had 13 domains (Table 5). The initial questions were generated within an international conference (4th International Lymphedema Framework Conference, June 2012, Montpellier, France) and the questions ranked in order of importance. The tool initially included 21 domains but this was reduced to 13 following the consultation process.

**Table 5.** The domains of the Core Tool

1. Type of facility in which data are collected	2. Demographics
3. Level of obesity	4. Mobility
5. Relevant comorbidities	6. Classification of lymphedema
7. Lymphedema history	8. Cellulitis History
9. Categories of treatment	10. Site of swelling
11. Wound area	12. Access to treatment
13. Subjective control of swelling	

The questions covered the most essential information required to understand the prevalence of chronic edema and its impact on health services. The tool was simple and rapid to complete. Translation and back translation of the core wound and swelling tools have been undertaken in Danish, French, Turkish, Italian, and Japanese thus adding another level of validation.

### Data fields within the core tool

The core tool identified key information about access to care and the types of treatment. This included whether any treatment was being given or not. For those receiving treatment, data were collected on the elements of complex decongestive therapy: skin care, exercise, manual lymphatic drainage, and types of compression. Other treatments noted included antibiotics, psychological support, and surgical treatments such as liposuction.

A body map was used to record the sites of swelling and the presence and nature of any concurrent wounds. Lower and upper mobility status was defined using a previously published classification.^9^ Information about the history of cellulitis and its treatment was recorded, including use of antibiotics and episodes requiring hospitalization. A WHO general category of weight was adopted due to the lack of available BMI for many, particularly those seen in community settings.^10^ Important comorbidities linked to chronic edema were also recorded.

The teams screening the patient were asked to make a subjective judgment about whether the swelling was controlled or not. While it is recognized that this question is open to professional interpretation, nevertheless this is an important issue that has previously been linked to whether patients are accessing treatment.^3^

Further questions examined whether chronic edema was a factor in determining or delaying discharge from hospital or was a reason for long-term community care. Issues of access to specialist treatment and the distance from services were also recorded.

### Expert review of the core tool

Each of the 8 participating frameworks engaged 10 professionals (generalists and specialists) to review the core tool using a set of 5 validated case studies to complete the tool and decide the classification. Data were entered into the database and the results were compared by five independent lymphologists for accuracy of classification and data quality. Of the 400 complete case reviews, correct classification occurred in 387/400 (97%). The 13 cases in which classification differed related to the decision about whether the case was due to primary or secondary lymphedema. This is a well-known and valid difficulty for clinicians.

### Module tools

The Module Tools aimed to assess the functional and quality-of-life impact using validated tools where possible. Further clinical information about wounds and treatment (where present) and details of the severity of swelling were included. Module Tools covered the following areas:

### Demographics and disability (WHODAS 2.0)

This well-validated tool included questions that explored the patient's personal circumstances for example, housing, employment, and education. WHODAS 2.0 is a 12-item disability assessment schedule completed by the patient.^11^

### Quality of life (LYMQOL and EQ-5D and LFSQQ)

Quality of life was assessed with a combination of disease-specific and generic tools.

- *LYMQOL* is a validated condition-specific quality-of-life assessment instrument (it is not specifically validated for patients with lymphatic filariasis) that assesses the impact of lymphedema on the patient's everyday living and health-related quality of life. The tool is validated for lower and upper limbs.^12^
- *EQ-5D* is a generic quality-of-life instrument applicable to a wide range of health conditions and provides a simple descriptive profile and single index value for health status.^13^
- *LFSQQ (Lymphatic Filariasis-Specific Quality-of-Life Questionnaire)* is a condition-specific instrument for patients with lymphatic filariasis. The LFSQQ is intended for use for patients within filarial areas and the questionnaire was developed based on an Indian lifestyle.^14^

### Details of swelling

This tool provided further details about the swelling. Each study site elected one measurement technique, which was standardized for all patients.

### Limb circumference and volume measures

Standardized circumferential limb measurements using a tape measure were the most widely used and accessible technique. Upper and lower limb circumference were measured bilaterally at two points according to the site of swelling:

○ mid-upper arm (10 cm proximal to the olecranon process)
○ forearm (10 cm distal to the olecranon process)
○ mid-thigh (20 cm proximal to the patella)
○ calf (20 cm distal to the patella)

### Limb volume methods (specialist centers)

Limb volume measures were undertaken in centers with access to specialist methods. Limb volume was expressed in milliliters for each limb using a standard method for all patients within the study site. The methods included water displacement and Perometry.

### Kaposi–Stemmer sign

The Kaposi–Stemmer sign involved pinching a skin fold at the base of the second toe or middle finger of the limb that had edema.^15^ The sign was judged to be positive if the skin could not be lifted and negative if the skin could be lifted normally.

Collectively, these data were used to describe and classify severity using the International Society of Lymphology staging tool^16–18^:

- ISL stage I: Early onset of the condition, where there is accumulation of tissue edema that subsides with limb elevation. The edema may be pitting at this stage.
- ISL stage II: Limb elevation alone rarely reduces swelling and pitting is manifested.
- ISL stage III: The tissue is hard (fibrotic) and pitting is absent. Skin changes such as thickening, hyperpigmentation, increased skin folds, fat deposits, and warty overgrowths develop.

### Wounds

This tool provided data about all wounds, including type of wound, location, severity, wound area, wound duration, history of infection, and frequency of dressing changes.

- Cancer

A specific tool for patients whose chronic edema was the consequence of cancer treatment or a direct effect of the disease was developed. This was a 14-domain questionnaire focusing on types of cancer and surgical and nonsurgical treatments. The types of cancer included were:

**Table:** 

Breast	Cervical
Endometrial	Ovarian
Bladder	Vulval
Colorectal	Melanoma
Head and neck	Other cancers

### Phase 1. Stage 4. Development and validation of a classification for chronic edema

A 15-member expert panel reviewed and assessed a set of eight case studies for content and face validity and to ensure that they reflected the chronic edema classification categories contained in the core tool. There was a 100% response rate. Five of the eight case studies achieved a 90% agreement level for the classification of lymphedema and these five case studies were used for the review of the core tool in stage 4.

The classification was made on whether the chronic edema was of primary origin or due to secondary causes (Table 6). In those with a secondary cause a number of key suspected contributory factors were included, such as venous disease and obesity. During the studies specialist lymphology teams undertook this classification where patients had been identified and confirmed to have chronic edema.

**Table 6.** Classification of Chronic Edema

Primary Lymphedema	
Secondary Lymphedema	Cancer
	Noncancer
	Contributory factors: venous disease, obesity, immobility, other (free text)

### Stage 5. Interrater reliability studies

The LIMPRINT study included a number of interrater reliability studies to assess the accuracy of detection of chronic edema using the pitting test and the other methods of clarification.

Dai et al.^7^ undertook a cross-sectional study within a long-term care hospital in Japan. The interrater reliability of the pitting test for evaluating edema was tested for 34 locations of the body. The pitting test was applied for 10 seconds with a similar force to that used by the expert assessor who acted as a “gold standard.” Detection of the presence of edema on removal of pressure was assessed using the modified Fukazawa method^19^ described in Table 7.

**Table 7.** Modified Fukazawa Method of Assessment for Detection of Pitting Edema

*Grade*	*Criteria*
0	There is no impression (no edema present)
1	Impression of the outline of the dimple is slightly differentiated by release of pressure and sometimes appears to be absent
2	Impression does not become clear at the beginning of pressure but occurs with further pressure and an impression is left after release
3	Deep impression remains after release of pressure that is clear on visual inspection at initiation of pressure
Nonpitting Edema (added to method)	Indentation made by pressure does not persist (nonpitting edema as seen in patients with hard tissue)

Five bedridden patients were assessed by the gold reference and four independent assessors. Agreement among the assessors was high at >0.85 with the kappa coefficient showing fair agreement (range 0.51–0.81) (Table 8).

**Table 8.** Interrater Reliability Results from Japan

*Rater ID*	*Agreement rate with “gold standard”*	*Cohen's kappa coefficient*
1	0.88	0.51
2	0.90	0.60
3	0.94	0.81
4	0.88	0.51

The same methodology was used in three U.K. studies. Two of the studies involved patients seen by community nursing services and one with patients within a residential care facility. Results from these studies confirmed a high rate of detection of chronic edema although mild edema was missed by a proportion of community nurses compared with the expert assessor.

### Phase 1. Stage 6. Construction and testing of the online database

#### Security and international requirements of the online data base

LIMPRINT data were managed by an electronic data management system that had a comprehensive set of security features that included the storage of data in encrypted form and individualized password-controlled access. The system had an audit trail, which tracked all activity on the system, including changes to data. As an added security feature, the user was automatically logged out after 10 minutes of inactivity, requiring a password to restart the application. An edit check function in the form of a Data Clarification Form was developed to check for flagging, missing, invalid, incomplete, or questionable data and required study-site clarification before the data were marked as complete. Individual sites had access to only their own data. The project manager and statisticians were able to access all data from the sites.

During the project, the data system was evaluated by the participating frameworks to ensure that it was fit for purpose and to access the training requirements for each site. Central training for all users was undertaken by ILF project manager and data management team.

#### Data transfer and compliance

The study faced important considerations relating to data protection and transfer of data. For countries within the European Union (EU), the EU Data Protection Directive guides such legislation places are restrictions on the exporting of confidential data to countries outside the EU. This includes the United States where the database was located. Within these restrictions the transfer of data was permitted provided three criteria were met:

- There was confirmation that the system complied with all EU Data Protection legislations and could ensure that sensitive health data were protected.
- The data were anonymized.
- Patients gave explicit consent to the transfer of their data to a country outside the EU.

In sites where transfer of data outside of Europe was prohibited due to research governance issues, the data were entered on to a password-protected data collection system using the same data capture fields and stored on secure servers.

### Phase 1. Stage 7. Development of an international protocol and sampling frameworks

The international protocol included a detailed description of the sampling procedures and study methods and was used in all sites.

### Methods for sampling in geographically based prevalence study

A comprehensive list was collated for each geographical area of all acute hospitals and relevant community health services that may meet people who have chronic edema. Examples of such services included:

○ General medical practitioner.
○ Specialist community medical practitioner.
○ Community nursing/home care nursing service.
○ Nursing home.
○ Elderly care residential home.
○ Specialist lymphedema service.
○ Acute hospital services (in-patient or/and outpatient).

A sampling frame was created, with the aim of the recruitment strategy to ensure that the widest range of patients were identified with different underlying conditions and comorbidities.

### Hospital prevalence studies

In hospital settings, a sampling framework of all wards and departments in which patients with chronic edema would be found was developed. The screening was undertaken in a single day or over several days in larger facilities. Trained staff screened all patients who consented to participate irrespective of their underlying disease or treatment regimen. Teams of researchers undertook the study with a lymphologist assigned to each team to undertake the chronic edema classification.

The bed capacity of each ward or unit was recorded, plus the number of beds occupied, the number of patients recruited, and the number excluded from consideration and the reasons for this. This enabled the prevalence to be calculated accurately in hospital settings.

### Community-based prevalence studies

All staff in relevant community health services were asked to approach each person on their existing case list to provide information and gain consent. All generalist practitioners participating in the study received training in the completion of the tools. The collection of data took place prospectively over a time frame, generally of 4 weeks and required all patients to be clinically screened and assessed. Numbers on community caseloads were recorded and those excluded from the study. The method of classification of chronic edema varied in different settings but included the use of specialist lymphedema therapist and tissue viability teams in some settings. In areas where this support was not available the more detailed classification was omitted (three studies in the United Kingdom).

### Random sampling techniques

A random sample was used in some population or facility-based studies, where a large number of patients with chronic edema was identified but additional modular tools were required. A random permuted block design allowed for a one third sample to be taken. Following completion of the core tool, the investigator checked the patient numbers of those identified with chronic edema against a pregenerated randomized list of sequential patient numbers to decide whether the patient was to be interviewed. Each clinical team held an individual randomization list.

### Specialist service profiles

LIMPRINT provided a unique opportunity to understand the profile of patients seen within specialist lymphedema and other types of services in different countries and to compare this with patients found in other settings. Patients gave informed consent for their inclusion in study sites, where ethics approval required this. The accuracy of the service lists was checked to ensure that it was up to date and discharge and deaths had been removed. Information on their edema status was based on their last visit and the type of lymphedema was taken from the clinical case records. As these patients were in specialist lymphedema services, the underlying stratification of primary and secondary lymphedema was already determined in addition to the comorbidities required for completion of the core tool.

### Phase 1. Stage 8. Development of a support manual and educational tools

LIMPRINT required the development of a range of educational materials to ensure that the data were collected accurately. This included a manual to supplement the protocol. Formal training standards were written that included a checklist of training and competency for all those collecting data. A video using reuseable learning outcomes was made to demonstrate how to undertake the pitting test, recognition of a positive stemmer sign, and fibrosis. All facility-based studies commenced with a half day training program that included checking of the first assessment by specialist practitioners. Data quality was enhanced by teams of researchers working in clinical areas, where any complex clinical questions could be confirmed before leaving the area.

### Phase 1. Stage 9. Establishment of quality control mechanisms

A range of quality control mechanisms was established.

### Preventing double counting

The prevention of double counting was essential to achieve an accurate prevalence. The double counting of patients was likely to take place within a geographical area when a patient might be identified by more than one service delivering care to the same patient or in an acute care setting if a patient moved departments. This was prevented by the allocation of a nonidentifiable number that was matched with a master identifier list of patients.

### Data quality checks

In addition to the quality checks within the electronic data system, additional checks were made of the paper-based data collection tools. Within each facility, quality monitors were established to check completeness of data from each clinical area before the research teams left the area and all forms were checked by a central coordinator. Similar mechanisms were established for community studies.

## Conclusion

The standardization of methods used in the LIMPRINT study provided a framework that allowed sites in different countries and from different facilities and types of health care systems to work together. The completion of LIMPRINT also demonstrated that an international initiative, which was supported by a strong ethos of partnership, could be undertaken. The results from the different studies will be discussed in a portfolio of articles.

The LIMPRINT team would like to thank all the sites for their contribution in generating the data for this study and their commitment to the ILF.
